# Physical child abuse demands increased awareness during health and socioeconomic crises like COVID-19: A review and education material

**DOI:** 10.1080/17453674.2020.1782012

**Published:** 2020-06-23

**Authors:** Polina MARTINKEVICH, Lise Langeland LARSEN, Troels GRÆSHOLT-KNUDSEN, Gitte HESTHAVEN, Michel Bach HELLFRITZSCH, Karin Kastberg PETERSEN, Bjarne MØLLER-MADSEN, Jan Duedal RÖLFING

**Affiliations:** 1 Department of Orthopaedics, Aarhus University Hospital; 2 Danish Paediatric Orthopaedic Research; 3 Research Unit for Mental Public Health, Department of Public Health, Aarhus University; 4 Department of Paediatrics, Aarhus University Hospital; 5 Department of Radiology, Aarhus University Hospital; 6 Department of Clinical Medicine, Aarhus University; 7 Corporate HR, MidtSim, Central Denmark Region, Denmark

## Abstract

**Background and purpose:**

Physical abuse of children, i.e., nonaccidental injury (NAI) including abusive head trauma (AHT) is experienced by up to 20% of children; however, only 0.1% are diagnosed. Healthcare professionals issue less than 20% of all reports suspecting NAI to the responsible authorities. Insufficient knowledge concerning NAI may partly explain this low percentage. The risk of NAI is heightened during health and socioeconomic crises such as COVID-19 and thus demands increased awareness. This review provides an overview and educational material on NAI and its clinical presentation.

**Methods:**

We combined a literature review with expert opinions of the senior authors into an educational paper aiming to help clinicians to recognize NAI and act appropriately by referral to multidisciplinary child protection teams and local authorities.

**Results:**

Despite the increased risk of NAI during the current COVID-19 crisis, the number of reports suspecting NAI decreased by 42% during the lockdown of the Danish society. Healthcare professionals filed only 17% of all reports of suspected child abuse in 2016.

**Interpretation:**

The key to recognizing and suspecting NAI upon clinical presentation is to be aware of inconsistencies in the medical history and suspicious findings on physical and paraclinical examination. During health and socioeconomic crises the incidence of NAI is likely to peak. Recognition of NAI, adequate handling by referral to child protection teams, and reporting to local authorities are of paramount importance to prevent mortality and physical and mental morbidity.

Physical abuse of children, i.e., non-accidental injury (NAI) including abusive head trauma (AHT), is experienced by up to 20% of children; however, only 0.1% are diagnosed with the ICD-10 code: T74.1 physical abuse (Christoffersen 2010, Stoltenborgh et al. 2013, Oldrup et al. 2016).

During the current COVID-19 crisis some European countries have reported an alarming increase in domestic violence by one-third (Delaleu 2020). Likewise, the risk of NAI is heightened during health and socioeconomic crises (Baird 2020, Peterman et al. 2020). Therefore, a Joint Leaders’ statement by the World Health Organization, UNICEF, Save the Children International, and SOS Children’s Villages International among others, highlights the acute risk of violence against children due to COVID-19 and calls for increased awareness (World Health Organization 2020).

The vast majority of NAI is reported by staff working at institutions (daycares, kindergartens, schools), which are temporarily closed during the COVID-19 pandemic. Healthcare professionals issue less than 20% of reports regarding suspected maltreatment to the responsible child protection authorities (Christoffersen 2010, Oldrup et al. 2016). Failure to recognize NAI due to insufficient knowledge among healthcare professionals may partly explain this low percentage (Villadsen et al. 2015).

Healthcare professionals need to be aware of the increased risk of NAI during COVID-19 and future health and socio-economic crises in order to act appropriately based on current knowledge of the issue. Only then can they begin to recognize patterns of NAI from the medical history and objective findings, and act appropriately through immediate consultation and referral to multidisciplinary child protection teams, who can clarify the suspicion and ensure child protection.

## Methods

Data were synthesized from a literature review, scholarly reports from Danish national authorities, and expert opinions on the clinical presentations of physical abuse, i.e., NAI including AHT, with the aim to provide educational material to encourage peer-to-peer teaching and facilitate a visual reference.

### Ethics and conflict of interests

This review complies with the Helsinki Declaration. The authors declare no conflicts of interest. Figures and Table are available (see also Supplementary data) for usage or modification according to a Creative Commons license (CC BY-SA 4.0) as long as this paper is attributed. The original, editable files can also be requested from the corresponding author.

## Results

### NAI clinical presentation and reporting

Physical abuse of infants and toddlers often presents to healthcare professionals as injury or illness such as seizures or respiratory distress (Table 1). In general, any injury in non-ambulatory children should raise suspicion and be discussed with a child protection team in order to plan the appropriate measures.

If the suspicion is raised, immediate referral and hospitalization is required to clarify the potentially lethal suspicion and ensure child protection.

In older children who are able to walk and talk, institutions (daycare, schools, sport clubs) report the majority of suspicions regarding child maltreatment to the local authorities and the police. However, healthcare professionals also need to stay alert regarding this age group.

### Red flags in the medical history and clinical presentations of NAI

It is important to consider the age and the developmental status of the child when distinguishing accidental injuries from NAI. Any injuries in an infant < 6 months are suspicious (Sugar et al. 1999, Maguire et al. 2005). Also, there is a need to be aware that NAI may present as part of the presentation of poly-victimization, a term referring to having experienced multiple victimizations of different kinds, e.g., sexual abuse, physical abuse, and neglect (Finkelhor et al. 2007). Children with any history of maltreatment should therefore be scrutinized for signs of NAI.

The key to increasing the relatively low percentage of healthcare-reported cases is to recognize NAI upon clinical presentation by considering suspicious findings revealed through the medical history, plus physical and paraclinical examinations. Risk indicators concerning the child, caregiver, and environment may be used as a supplement to guide clinical attention, although one should be aware of detection bias when judging already struggling families without sufficient clinical evidence (Widom et al. 2015) (Figure 1, see Supplementary data).

Child protection is paramount and barriers that hamper reporting must be overcome, even if this might stigmatize families until the suspicion has been fully investigated. For instance, the most common orthopedic injury in abused children is a fracture of the femur or humerus (Figure 2, see Supplementary data) (Loder and Feinberg 2007). NAI is diagnosed in about 25% of these fractures, when occurring under the age of 2 years. Hence, in the majority of cases, highly-specific fractures/injuries are absent and the combination of history and injuries should alarm the physician (Loder and Feinberg 2007, Kemp et al. 2008).

Maltreatment may present as disturbed sleep, unusual anger and irritability, withdrawal, attention and concentration difficulties, repeated and intrusive thoughts, helplessness, insecure relationship with caregivers, and intense emotional distress, particularly when confronted by trauma reminders (Al Odhayani et al. 2013). Also, it is important to recognize that children can exhibit problematic emotional and behavioral reactions long after the abuse or neglect has ended (Sege and Amaya-Jackson 2017). However, these potentially subtle changes in behavior may be difficult to detect during brief encounters such as emergency department visits.

### Skin manifestations

Skin manifestations are the most common findings in NAI, and present in up to 90% of victims of physical abuse (Kos and Shwayder 2006). Hence, physical examination of the entire body is mandatory. In general, skin manifestations are most frequently classified into bruises (Figure 2, see Supplementary data), bite marks, and thermal injuries. Oral injuries are often considered as a separate entity, and lack highly specific signs of NAI, thus warranting an odontological opinion.

It can be difficult to distinguish accidental injuries from NAI, but considering the location, pattern, and the age of the child at the time of injury will provide a clue to the suspected type of injury. Equally important is to consider underlying skin diseases, either as a coexisting condition or as a differential diagnosis.

#### Bruises

Bruising is the most common skin manifestation, and it is often inflicted by blunt trauma leaving a pattern marking (Swerdlin et al. 2007). In NAI bruises are often clustered on protected areas such as the Torso (e.g., chest, abdomen, back, buttocks, genitourinary region, and hip), Ears and Neck (TEN) (Maguire et al. 2005, Pierce et al. 2010). TEN bruising in a child < 4 years or any bruising in a child < 4 months has a sensitivity of 95% and specificity of 84% for prediction of abuse (Pierce et al. 2010). Abdominal bruising is rare, but warrants investigation of the internal organs, as 10% of victims will have intra-abdominal injury (Sheybani et al. 2014).

Bruises from accidents are located on bony prominences and occur more frequently in ambulatory children.

#### Bite marks

All bite marks are suspicious of abuse, and considered dangerous, as they can be a source of infection (Kos and Shwayder 2006).

Consulting a forensic odontologist regarding the bite marks can be valuable, as such professionals possess various techniques to identify the perpetrator. Adult bites have an intercanine distance of > 3 cm, which can distinguish them from child and animal bites (Kos and Shwayder 2006).

#### Thermal injuries

Thermal injuries account for up to 20% of all child abuse cases and data from burn centers suggest that nearly 14% of burn injuries in children are due to abuse, with increased hospitalization time and mortality rate compared with cases of accidental injury (Peck and Priolo-Kapel 2002, Thombs 2008, Royal College of Paediatrics and Child Health 2018). The victims are often < 3 years. Distinction is made between immersion and contact injuries. Often the inflicted thermal injuries leave characteristic patterns that are highly suspicious of child abuse.

Immersion such as scalding with hot tap water is most common. This tends to create symmetrical and distinct lines of demarcation. Frequent mechanisms include holding the child’s hands and feet under water (glove-and-stocking pattern, sparing of the palm), or submerging the child in hot water in a flexed position, creating a so-called zebra pattern with sparing of the flexural creases including palms. In general, inflicted burn injuries cover a wider and deeper surface area, and tend to include rather the back, buttocks, perineum, and lower extremities with symmetrical and clear demarcation lines as compared with accidental burn injuries (Thombs 2008). The most frequently reported contact burns include those inflicted by cigarettes, while other instruments include iron, hairdryer, cigarette lighters, oil, flame and chemical burns (Royal College of Paediatrics and Child Health 2018).

### Radiological red flags

**Figure 3:**
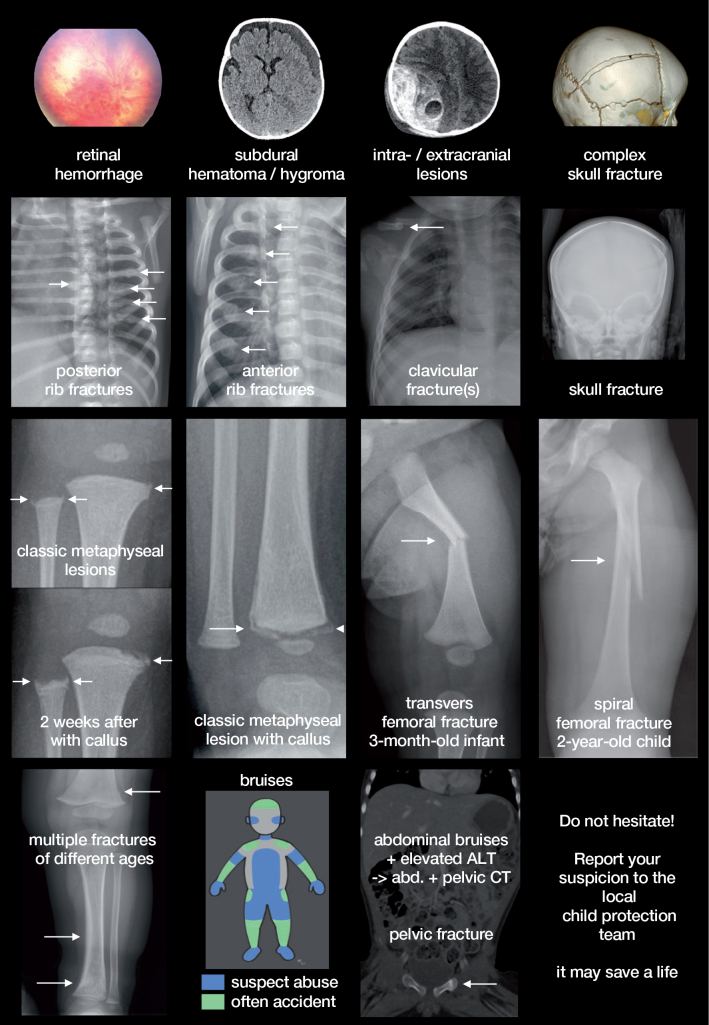
Radiological findings associated with NAI. While some findings are highly specific for NAI, the less specific findings are common in both NAI and accidents. Thus, we refrained from subdivision as any of these findings without an appropriate accident should result in involvement of a child protection team.

NAI represents a small proportion of all childhood fractures, but all healthcare professionals should be able to recognize the characteristics of fractures resulting from abuse (Figure 3). In infants and toddlers, physical abuse accounts for 12–20% of all fractures (Leventhal et al. 2008), thus indicating a skeletal survey in children < 24 months (Wootton-Gorges et al. 2017). Approximately 80% of all fractures caused by NAI occur in children < 18 months, and the proportion of fractures caused by child abuse declines rapidly as ambulatory function develops (Leventhal et al. 2008).

Importantly, the majority of physically abused children present with bruises and a simple fracture, which also can occur after accidents and thus has a lower specificity. Highly specific fractures for NAI and AHT are less common, but should be recognized upon presentation.

*Multiple fractures and fractures of different ages* should alert the clinician and any concern by the treating healthcare professional based on any anamnestic, objective, or radiological red flags and risk indicators should be considered in consultation with a child protection team.

### Common and highly specific fractures

*Classic metaphyseal lesions* (CML, i.e., metaphyseal corner and bucket-handle fractures) are the most specific and common signs of NAI in children < 18 months (Figure 3). They occur most commonly in the lower extremities around the knee and ankle, but are also seen in the upper limbs. Diaphyseal fractures are more common in ambulant children, but less specific (Kemp et al. 2008).

*Multiple rib fractures* can either be detected incidentally in a child presenting with respiratory compromise or on a skeletal survey on suspicion of NAI. Multiple rib fractures, especially of posterior location, have a high positive predictive value (Barsness et al. 2003, Kemp et al. 2008).

*Less common, but highly specific fractures* for NAI include epiphyseal separations, fractures of the digits, as well as complex skull fractures, while fractures of the scapula, sternum, and spinous processes are rare.

### Intracranial lesion

Subdural hematomas have been reported in up to 90% of young infants with AHT. Albeit not pathognomonic for AHT, they do become strongly suggestive of AHT when several SDH of different dates are observed, or the claimed injury mechanism is incompatible with, for instance, a simple fall from less than 1.5 m. Other types of intracranial hemorrhages may also be suggestive of AHT but are also common after accidents. Parenchymal injury is the most significant cause of morbidity and mortality (Choudhary et al. 2018)

### Abusive head trauma (AHT)

AHT accounts for around 50% of severe traumatic head injury cases (Keenan et al. 2003), and it is the leading cause of the death in children < 2 years with a peak before 5 months (Maguire et al. 2011). It is important to remember that no single AHT injury has intrinsic diagnostic value, therefore all findings and history should be considered together. Kelly et al. (2015) found that in children < 2 years the characteristics of AHT included hypoxic-ischemic injury (97%), no history of trauma (90%), no external evidence of impact to the head (90%), subdural hemorrhage (89%), and complex skull fractures with intracranial injury (79%).

In recent years, several clinical prediction or decision rules for AHT have been developed to differentiate between AHT and other reasons for a validated intracranial injury and likewise have a high sensitivity and positive predictive value (PPV) for AHT (Pfeiffer et al. 2018). These comprise tools such as the Pittsburgh Infant Brain Injury Score (PIBIS), which aids the decision to perform head CT scans in well-appearing children under 1 year in the emergency department (sensitivity 93%), while the Pediatric Brain Injury Research Network’s (PediBIRN) and Predicting Abusive Head Trauma (PredAHT) have been developed to differentiate between AHT and other reasons for a validated intracranial injury and have a high sensitivity and PPV for AHT.

Predicting Abusive Head Trauma (PredAHT) found a positive predictive value (PPV) of 85% for AHT when intracranial hemorrhage in children < 3 years was accompanied by at least 3 of 6 key features (head/neck bruising, seizure, apnea, rib- or long-bone fractures, and retinal hemorrhage). PediBIRN’s AHT probability calculator uses 4 clinical parameters: (1) “clinically significant respiratory compromise at the scene of injury, during transport, in the emergency department, or prior to hospital admission”, (2) “bruising of the torso, ear(s), or neck,” (3) “subdural hemorrhage or fluid collection that is bilateral or that involves the interhemispheric space,” (4) “any skull fracture(s) other than an isolated, unilateral, non-diastatic, linear, parietal, skull fracture.” The clinical feasibility of these tools and the perceived disadvantages, i.e., possible over-reliance and false reassurance are going to be investigated (Pfeiffer et al. 2018). Nonetheless, clinicians may benefit from applying these tools or at least being aware of the key clinical findings that these tools take into consideration.

## Discussion

The initiative for this study was triggered by the alarming surge of domestic violence precipitated by the restrictions imposed to contain the COVID-19 pandemic (Human Rights Watch 2020).

Peterman et al. (2020) identified distinct pathways for how pandemics might increase violence against intimate partners and children: “(1) economic insecurity and poverty-related stress, (2) quarantines and social isolation, (3) disaster and conflict-related unrest and instability, (4) exposure to exploitative relationships due to changing demographics, (5) reduced health service availability and access to first responders, (6) inability to temporarily escape the abuser, and (7) virus-specific sources of violence.”

In attempting to understand the surge in risk, it is widely accepted that stressors overcoming supportive factors comprise the underlying etiology explaining the majority of physical abuse (Belsky 1993). Earlier studies have shown increased risk of physical child abuse as a consequence of society-wide stress exemplified by natural disasters (Keenan et al. 2004, Melissa 2012). Stressors such as poverty (Berger et al. 2011, Doidge et al. 2017), unemployment (Krugman et al. 1986), and parental physical and mental health (Chang et al. 2018) have been shown to increase the risk of physical child abuse—all of which are likely to be affected by the current situation. In addition, the inability to escape the perpetrator due to restrictions of movement might further aggravate the risk (Peterman et al. 2020).

However, the scientific evidence regarding the association of socioeconomic health crises and NAI varies in methods and their applicability to the current situation, and the incidence of NAI is difficult to access objectively during times of crises. During previous Ebola epidemics an increase in NAI and child abuse was reported in the affected countries (Kostelny et al. 2016). The lessons learned from these crises regarding child protection (in developing countries) were published by UNICEF (UNICEF 2016).

During the first month of the confinement in France, the police received more reports of domestic violence and intervened in 92 child abuse cases; helplines received around 20% more reports of child abuse, via either the victims, relatives, or their network (Innocence En Danger 2020). Conversely, the Canadian regional social services received 75% fewer daily notifications of suspected child abuse (ICI.Radio-Canada.ca 2020). A similar tendency was observed by the Danish authorities, who reported a 42% decline in notifications regarding suspected child abuse immediately after the “lockdown” of society that included daycares and schools (Scheel et al. 2020).

Nearly all of the prevailing tools to mitigate the impact of domestic violence are based on the social environment, assistance, and access to healthcare. Due to confinement measures, closing of schools, kindergartens, and daycares, and the accumulating pressure on healthcare systems these tools are no longer readily available, and thus the risk of NAI will increase. Notably, the stress exhibited on the healthcare system challenges frontline healthcare professionals’ ability to maintain the usual standards of care. This raises serious concerns that NAI, which is already a hidden but frequent problem, risks being left unnoticed and unsuspected, leading to higher child morbidity and mortality as well as long-term negative developmental consequences (Buckingham and Daniolos 2013). NAI has been studied as one of several adverse childhood experiences (Felitti et al. 2019), and has been shown to carry increased risks of future ischemic heart disease (Gilbert et al. 2015), cancer, alcoholism, depression, suicide attempts (Felitti 1998), post-traumatic stress disorders (Cross et al. 2018), and social phobia (Scott et al. 2010). In addition to the direct consequences for the victim, child abuse bears high economic costs (Peterson et al. 2018).

The solution to the hidden problem of NAI requires a multidisciplinary, multilateral, and multistep approach (prevention, detection, and intervention). The complexity and universal pertinence of the problem is so extensive that it has been listed on the EU’s 2030 Agenda for the 17 Sustainable Development Goals proposed by the UN (UN 2015).

Medical professionals are often presented for the victims of NAI in an emergency setting, leaving a narrow window for detecting and facilitating appropriate follow-up of NAI.

A global meta-analysis (Stoltenborgh et al. 2013) concludes that physical abuse is 75 times higher than suggested by official reports. Consulting different hospitals after trauma instead of making recurrent visits to the same hospital, and frequent changes of primary healthcare provider (Friedlaender et al. 2005), as well as any disabilities of the child, might either conceal or delay the diagnosis (Nowak 2015). Furthermore, numerous barriers to reporting NAI exist, which partly explain the underreporting: insufficient knowledge concerning NAI and thus failure to recognize it, and delayed or inappropriate decision-making during the diagnostic process.

Many definitions of NAI exist in the legal and scientific literature, but there is no consensus on an absolute definition. For further information on terminology, Medical Subject Headings and ICD-10 codes please refer to Supplementary data, Table 1. We advocate the use of ICD-10 codes: physical abuse in conjunction with non-accidental injury and abusive head trauma (Choudhary et al. 2018).

In conclusion, healthcare professionals should be aware of the heightened risk of physical abuse of children during health and socioeconomic crises. Only if healthcare professionals are familiar with NAI—through the anamnestic, objective, and radiological red flags and risk indicators—can they recognize NAI and act appropriately by consulting with the multidisciplinary child protection team who can verify or reject this devastating diagnosis. In addition, any child considered in immediate danger of further harm, and all children less than 2 years old, should be hospitalized to provide shelter and enable further investigations. We hope that this review and its illustrations can help frontline healthcare staff to achieve this aim and potentially save lives or minimize the long-term effects of adverse childhood experiences.

### Supplementary data

Supplementary Figures: “All figures from the current manuscript for free usage and modification,” and Supplementary Table: “Terminology, Medical Subject Heading of NAI” are available as supplementary data in the online version of this article, http://dx.doi.org/10.1080/17453674.2020.1782012

## Supplementary Material

Physical child abuse demands increased awareness during health and socioeconomic crises like COVID-19

## Notes

PM, LLL, and TGK contributed equally to the study. They conducted the literature search and drafted the first version of the manuscript. GH contributed with clinical cases, photos, and critical revision of the manuscript. MBH and KKP provided radiological images and radiological red flags. BMM contributed in drafting the manuscript and critical revision. JDR contributed to all parts of the study and layout of the fig

The authors would like to express their gratitude to Rikke Holm Bramsen, Børnehus Midt, Muncipality of Aarhus, Denmark and Ole Ingemann Hansen, Department of Forensic Medicine, Aarhus University and Hans Ulrik Møller, Department of Ophtalmology, Aarhus University Hospital for their invaluable contributions.

*Acta* thanks Nanni Allington and Hanne Hedin for help with peer review of this study.
