# Physical child abuse demands increased awareness during health and socioeconomic crises like COVID-19: A review and education material

**DOI:** 10.1080/17453674.2021.1975912

**Published:** 2021-09-10

**Authors:** Polina MARTINKEVICH, Lise Langeland LARSEN, Troels GRÆSHOLT-KNUDSEN, Gitte HESTHAVEN, Michel Bach HELLFRITZSCH, Karin Kastberg PETERSEN, Bjarne MØLLER-MADSEN, Jan Duedal RÖLFING

**Affiliations:** 1 Department of Orthopaedics, Aarhus University Hospital; 2 Danish Paediatric Orthopaedic Research; 3 Research Unit for Mental Public Health, Department of Public Health, Aarhus University; 4 Department of Paediatrics, Aarhus University Hospital; 5 Department of Radiology, Aarhus University Hospital; 6 Department of Clinical Medicine, Aarhus University; 7 Corporate HR, MidtSim, Central Denmark Region, Denmark

The infographic about red flags that should raise suspicion of physical child abuse (Figure 1 in Supplementary data) has been updated with minor text corrections. Furthermore, a complete reference list for the infographic has been added.

## Figures and Tables

**Figure 1:**
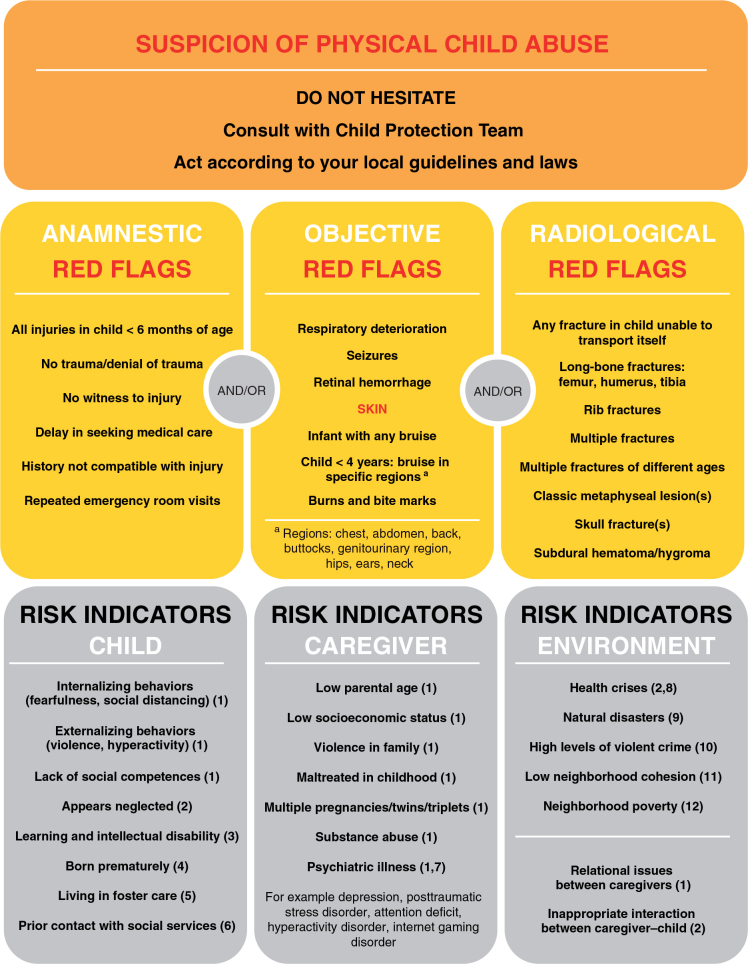
Red flags and risk indicators of physical child abuse.
