# Supplementary material for: Physical child abuse demands increased awareness during health and socioeconomic crises like COVID-19: A review and education material
Source: Acta Orthop. 2020 Jun 23;91(5):527–33. doi: 10.1080/17453674.2020.1782012 (PMC8023935; doi:10.1080/17453674.2020.1782012)
Supplement: Physical child abuse demands increased awareness during health and socioeconomic crises like COVID-19 [file IORT-91-1782012-s001.pdf]

Supplementary data

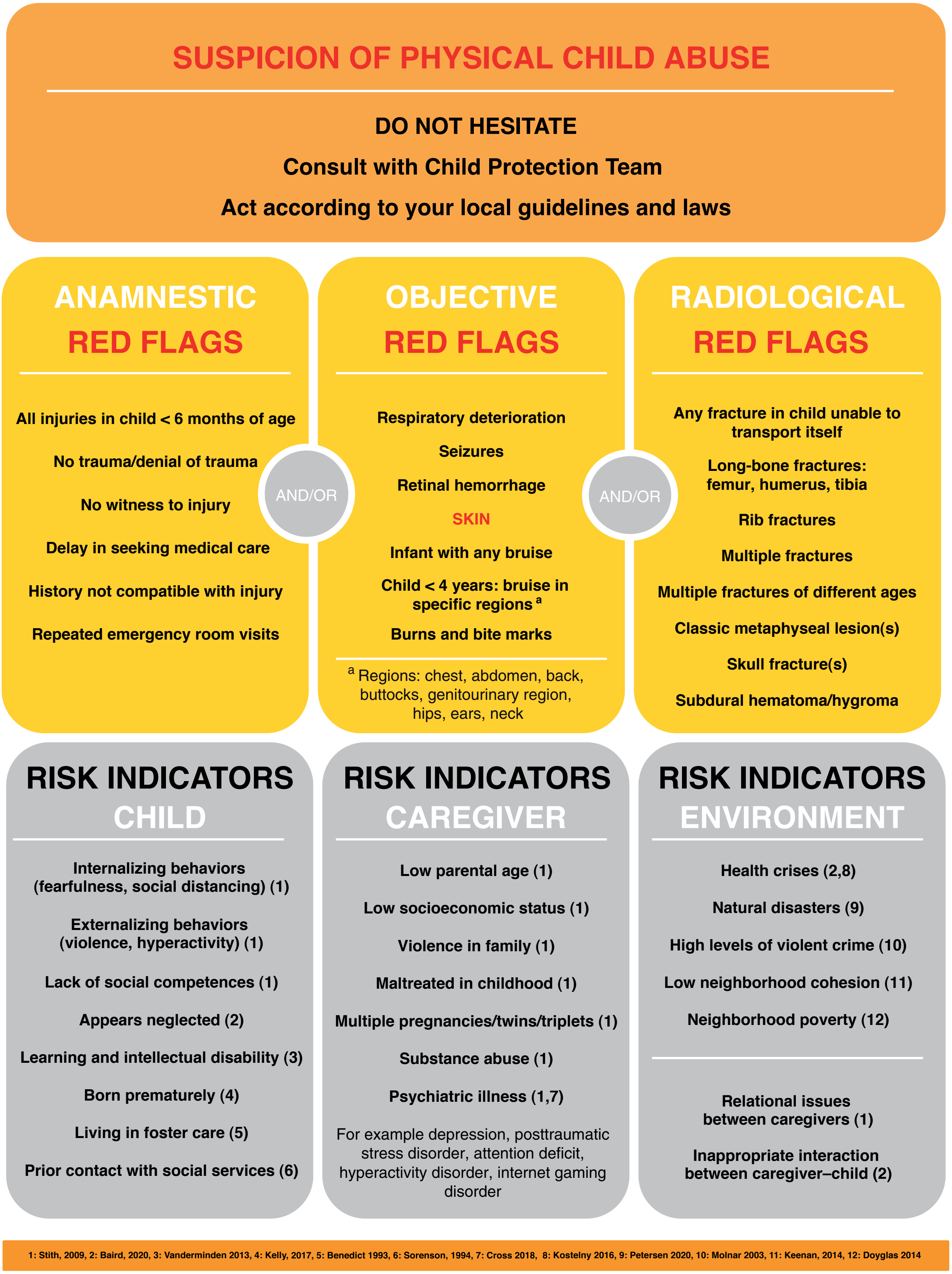

Figure 1. Red flags and risk indicators of physical child abuse.

 Acta Orthopaedica 2020; 91 (DOI: 10.1080/17453674.2020.1782012)

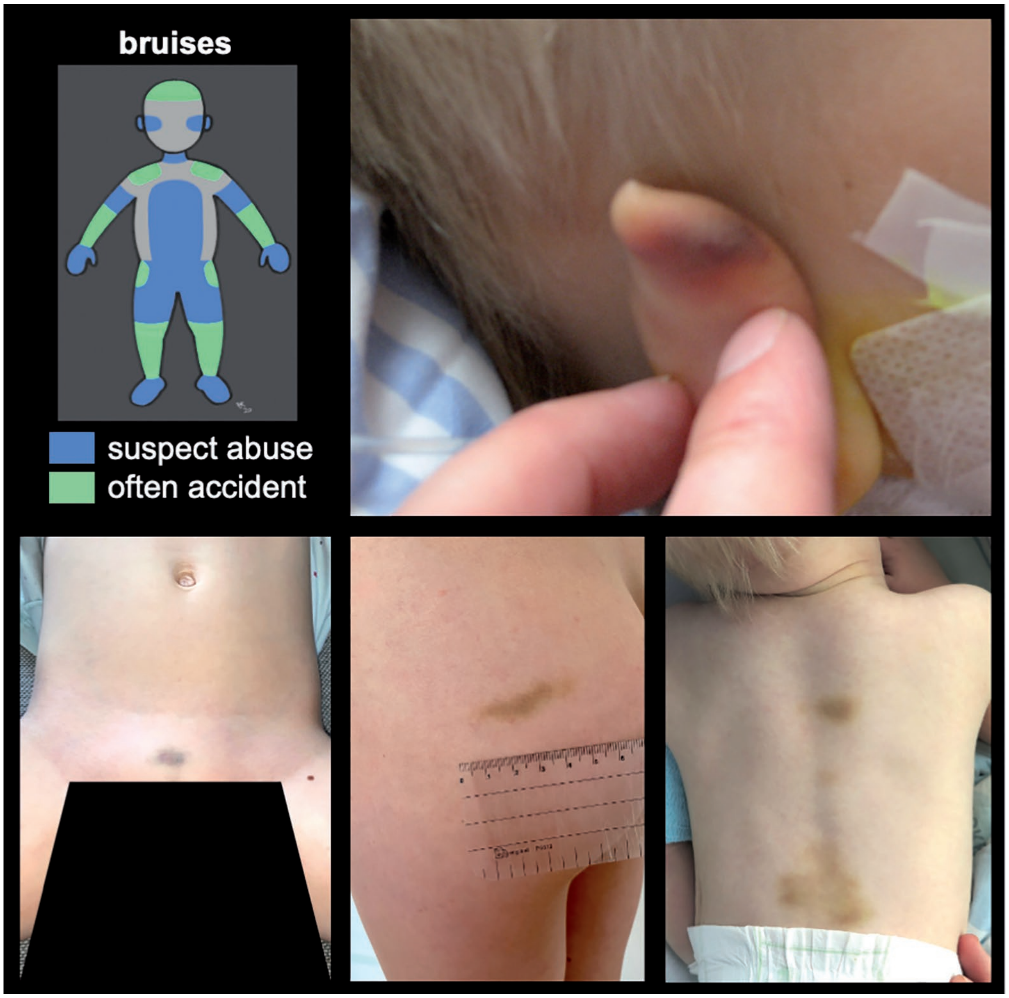

Figure 2. Bruises are the most common finding in physically abused children, especially when present in the blue areas and occurring in children under 4 years of age. In particular, bruising of the torso, ears and neck (TEN region) is highly suspicious of NAI. Physical examination of the entire body is mandatory

Clinical presentation of NAI illustrating how red flags and risk indicators should raise attention

History and clinical presentation	Findings after clinical and paraclinical assessment by the child protection team
A 5-month-old infant was brought to the emergency department as a result of seizures with no history of trauma. Medical officer found bruises to the back and a CT scan revealed bilateral frontal subdural hemorrhages (SDH).<br>[Bruises had been noticed by the grandparents on several occasions, but had not been reported].	– Bilateral retinal hemorrhages<br>– CML (distal femur and tibia undergoing healing)<br>– Signs of older bilateral fractures of several costae<br>– Elevated liver enzymes (ALT 129)<br>– Kidney abnormalities, possibly sequelae after trauma<br>– Signs of old fracture of the pubic bone (ramus superior)
An unresponsive 10-month-old was brought to the emergency department. The mother was out shopping for groceries and the father said he heard a bump from the nursery, while he was playing a video game online. He found his child unconscious next to the bed.<br>A CT scan revealed bilateral subdural hematomas and a complex occipital skull fracture.	– Truncal bruising<br>– Multiple rib fractures<br>– Retinal hemorrhage<br>– CML (distal femur)
A 2.5-year-old child presented with an acute diaphyseal femoral fracture, accompanied by his mother. He had had 2 previous visits without fractures. According to the mother he had just learned to climb and fell in a playground. Due to the number of hospital visits and the spiral nature of the femoral fracture the child protection team was involved.	During hospitalization, adequate parental skills and normal development and behavior of the child were observed. Furthermore, the skeletal survey did not reveal additional injuries.<br>The highly relevant initial suspicion of child maltreatment was disproved.
A 12-year-old boy presented in school with bruising on his back, with a pattern indicating use of a belt and extension cord.<br>The boy had recently started in school after 12 months of absence along with his 3 younger siblings.<br>The family had frequently moved between several municipalities.<br>A gym teacher notified the social authorities.	Poly-victimization (neglect, physical and sexual abuse):<br>– Bruises and scarring on the back, upper arms and neck<br>– Signs of caries and dental neglect<br>– Signs of forced oral sex with injuries on tongue and lip ties and oral gonorrhea infection<br>– Inadequately treated asthma<br><br>All 4 siblings had incomplete compliance with vaccination program, signs of malnutrition, vitamin D deficiency, pinworms, and lice.
